# Low adherence to the guideline for the acute treatment of migraine

**DOI:** 10.1038/s41598-022-12545-2

**Published:** 2022-05-19

**Authors:** Astrid Olesen, Henrik Winther Schytz, Sisse Rye Ostrowski, Mie Topholm, Kaspar Nielsen, Christian Erikstrup, Susan Mikkelsen, Ole Birger Pedersen, Jes Olesen, Thomas Folkmann Hansen, Mona Ameri Chalmer

**Affiliations:** 1 Department of Neurology, Danish Headache Center, Copenhagen University Hospital, Rigshospitalet, Denmark; 2 Department of Clinical Immunology, Centre of Diagnostic Investigation, Rigshospitalet, Denmark; 3 Department of Clinical Medicine, University of Copenhagen, Copenhagen, Denmark; 4 Department of Clinical Immunology, Odense University Hospital, Odense, Denmark; 5 Department of Clinical Immunology, Aalborg University Hospital, Aalborg, Denmark; 6 Department of Clinical Immunology, Aarhus University Hospital, Aarhus, Denmark; 7 Department of Clinical Immunology, Zealand University Hospital, Køge, Denmark; 8 Novo Nordic Foundation Center for Protein Research, Copenhagen University, Copenhagen, Denmark

**Keywords:** Patient education, Migraine

## Abstract

The real-world use of triptans in the treatment of migraine is disappointing. Only 12% of the Danish migraine population purchased a triptan between 2014 and 2019, and only 43% repurchased a triptan after first prescription. The aim of the present study was to assess whether physicians and patients adhere to the therapeutic guideline on acute migraine treatment. We interviewed 299 triptan experienced participants with migraine and 101 triptan naïve participants with migraine from the Danish Migraine Population Cohort, using a semi-structured questionnaire. Descriptive statistical analyses were used to study the association with triptan use and the assessed factors. Among triptan naïve participants with migraine, 64% had consulted their general practitioner about their migraine, of whom only 23% received information about the possibility of triptan treatment. Among triptan experienced participants, 77% had only tried one type of triptan. Only 12% could recall they had been informed by their general practitioner to try each triptan three times before giving up. Twenty percent were informed to try three different triptans in total, if the first did not work. In disagreement with the guideline, participants who reported a low pain reduction by a triptan had only tried one type of triptan. Our study shows a low adherence to therapeutic guideline for the attack treatment of migraine. There is a need for better education of general practitioners regarding treatment of migraine. Future campaigns should aim to inform both the public and the general practitioner about antimigraine treatments.

## Introduction

Effective acute migraine treatment is necessary to enhance patients’ quality of life. Recently, it was reported that only 12% of the Danish migraine population had purchased a triptan between 2014 and 2019^1^. Following their first purchase, only 43% repurchased a triptan within 5 years^1^, indicating a low persistence rate. This was surprising in a country with free headache care and access to several inexpensive generic triptans. Furthermore, a national clinical guideline has existed for several years on how to prescribe and use triptans as acute medication for migraine^2^. In other European countries, acute migraine treatment with triptans is even further from the expected^3^. Only 10–18% of migraine patients consult a physician, and adequate triptan treatment was only applied in 3–11%^4^. In Russia, 23% of headache patients consulted a physician and only 6% of migraine patients used triptans^5^. Clinical trials have proved that triptans are very effective and beneficial compared with placebo^6^. Effectiveness is defined by the therapeutic gain which is the response to treatment of active substance within two hours minus the response to placebo^2^, and oral triptans generally have a therapeutic gain of 35% after 2 h^7^. For pain freedom at two hours the therapeutic gain ranges between 16 and 35%^7^.

The minimal use of effective and inexpensive acute migraine drugs among migraine patients constitutes a paradox which requires attention. We therefore aimed to assess the adherence to the therapeutic guideline on acute migraine treatment among physicians and individuals with migraine. To that aim, we assessed the association between the use or non-use of triptans and relevant factors, using a semi-structured interview. We randomly selected participants with migraine from the Danish Migraine Population Cohort which is largely representative of the Danish migraine population^8^.

## Results

**Figure 1:**
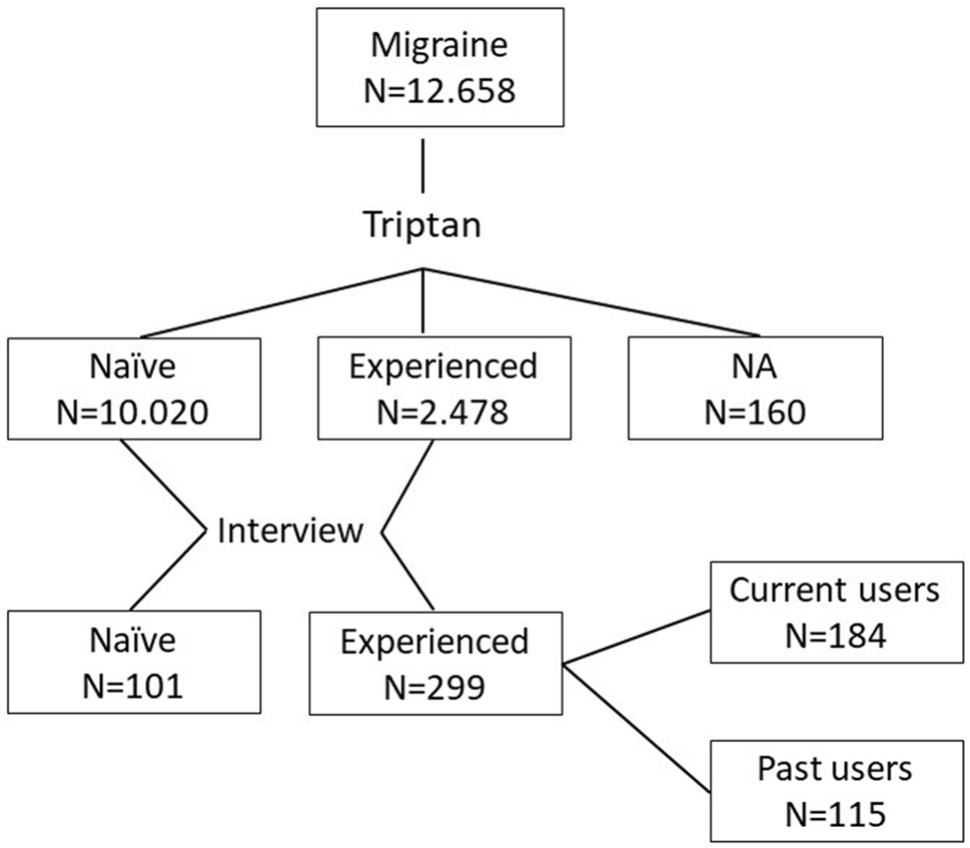
Inclusion of study participants from The Danish Migraine Population Cohort. Triptan naïve (N = 101) and triptan experienced (N = 299) participants were randomly recruited from the Danish Migraine Population Cohort.

Flow chart of inclusion of study participants is presented in Fig. 1. We interviewed 400 participants with migraine from DaMP, 74% women (N = 296) and 26% men (N = 104). The interview was stratified on participants who were triptan naïve (N = 101), i.e. had never tried a triptan, and participants who were triptan experienced (N = 299). Among triptan naïve participants, 46% (n = 47) fulfilled a migraine with aura (MA) diagnosis and 54% (n = 54) fulfilled a migraine without aura (MO) diagnosis. Among triptan experienced participants, 47% (n = 140) fulfilled a MA diagnosis and 53% (n = 159) fulfilled a MO diagnosis. Diagnoses were assessed according to the third edition of the International Classification of Headache Disorders^9^. If participants fulfilled both a MA and MO diagnosis, they were added to the MA group. The proportion of high frequency episodic migraine (HFEM)^10^ among triptan naïve participants was 3% (n = 3) and 6% (n = 17) among triptan experienced participants. For HFEM, there was no significant difference between triptan naïve and triptan experienced participants, p = 0.09 (Chi-sq). Among the triptan experienced participants, there was no significant difference between the participants who had terminated their triptan treatment (11% terminated, N = 13) and those who were still using triptans (9%, N = 16), p = 0.461912 (Chi-sq). The baseline characteristics are presented in Table 1.

**Table 1:** Baseline characteristics of participants with migraine.

Triptan	Naïve N	Experienced N
Total	101	299
Median age (IQR)	44.9 (16.4)	46.8 (18.5)
Women (%)	62 (62%)	234 (79%)
**Days with migraine within the last three months (N)**		
0	54	95
1–3	42	137
4–7	< 5	50
8 or more*	< 5	17

Baseline characteristics of triptan naïve and triptan experienced participants with migraine. According to the study protocol, results of number of participants between n = 0–4 must be indicated as n < 5. *IQR* inter-quartile range. *Defined as High Frequency Episodic Migraine.

### Triptan naïve participants

**Figure 2:**
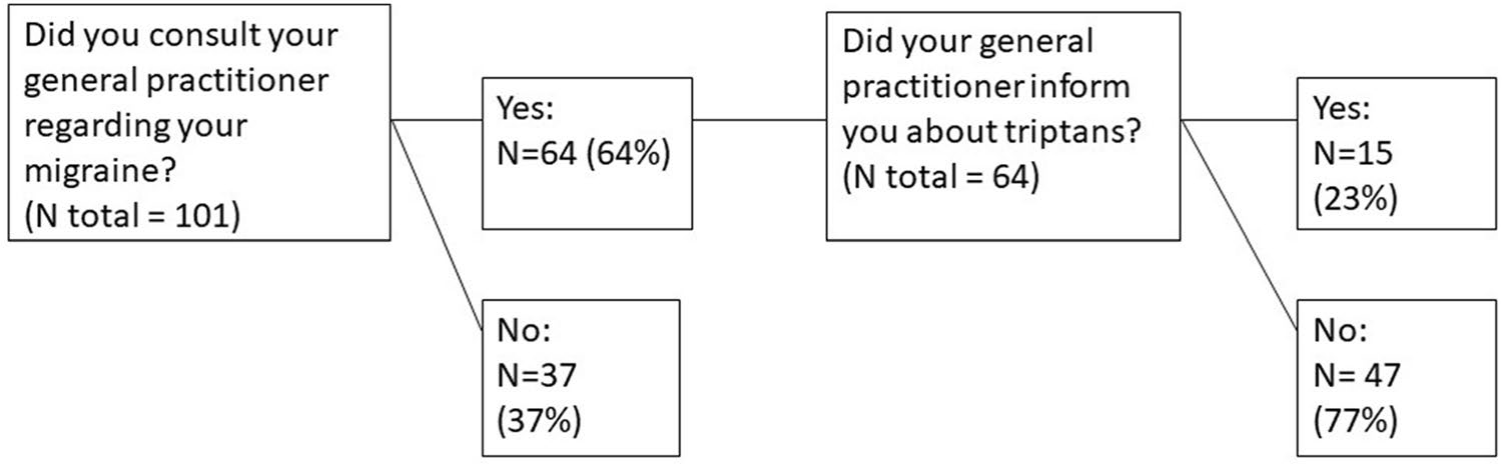
Flow chart of triptan naïve participants who had consulted their general practitioner regarding their migraine.

Eighty percent of participants in DaMP were triptan naïve. Among triptan naïve participants, 64% had consulted their GP about their migraine. Only 23% received information about triptans (Fig. 2), and 89% used other treatment for their migraine. In total, 80 participants used simple analgesics (paracetamol, ibuprofen) and combination analgesics (aspirin/caffeine and aspirin/codeine combinations), see Supplementary Table 1. Ten participants reported taking other treatments, which did not fall into these categories. The most used treatments were a combination of paracetamol and ibuprofen (N = 27), paracetamol alone (N = 21), and combination analgesics (paracetamol, ibuprofen or aspirin/caffeine) (N = 10). Five participants used paracetamol, ibuprofen, aspirin/caffeine or aspirin/codeine (Supplementary Table 1). The triptan naïve participants were asked to evaluate their level of function two hours post intake of acute treatment, compared with their level of function pre intake. Eighty-four percent reported that their level of function was improved to half of normal or more, while 16% reported a poor improvement of function. Furthermore, 83% reported a pain reduction of ≤ 50%, two hours post intake (Table 2).

**Table 2:** Effect of acute treatment for migraine attack two hours post intake.

	Triptan naïve N (%)	Triptan experienced N (%)
**Function level**		
Increased to ≥ 50%	85 (84)	220 (74)
Significantly impaired or no effect at all	16 (16)	78 (26)
**Pain reduction**		
≤ 50%	75 (83)	55 (18)
> 50%	15 (17)	243 (82)

Effect of acute treatment other than triptan among triptan naïve participants and effect of triptans among triptan experienced participants.

The majority of the triptan naïve participants reported that their migraine attacks were not severe enough to use triptans (60%). Others reported a general aversion towards medicine (15%), while 12% were satisfied with other treatments and did not wish to try triptans. Finally, 2% were concerned about adverse events.

### Triptan experienced participants

Among the 299 triptan experienced participants, 97% (N = 291) had consulted their GP regarding their migraine and 62% (N = 184) were still using triptans. As recommended by the clinical guideline, 73% (95/130) of the participants who had recurrent migraine were instructed by their GP to take an additional dose at least two hours after the first dose if the migraine recurred within 24 h. However, only 12% (N = 36) were informed to try the same triptan at least three times before concluding that the triptan was not effective. Likewise, only 20% (N = 60) were informed that they should try three different types of triptans, if the first or second choice was not effective or if there were adverse effects (Table 3).

**Table 3:** Information received by the general practitioner (GP) among triptan experienced participants.

Information about migraine and treatment received by GP	Yes N (%)	No N (%)
Migraine diagnosis	291 (97)	8 (3)
Migraine treatment	292 (98)	7 (2)
Treatment for recurrent migraine	95 (73)	35 (27)
Try the same triptan at least 3 times	36 (12)	263 (88)
Try three different types of triptan	60 (20)	239 (80)

Information by the general practitioner (GP). Detailed questions about information received by the GP regarding diagnosis and the GP’s guidance regarding triptan treatment were only given triptan experienced participants. Moreover, the question regarding information about treatment for recurrent migraine, was only given to participants with recurrent migraine within 24 h (N = 130).

Seventy-seven percent (N = 230) had tried one type of triptan, of whom, 54% did not wish to try another triptan. The majority of participants had tried Sumatriptan (N = 184). Twenty percent (N = 59) had tried two types of triptans, and the second choice of triptan was most commonly Rizatriptan (N = 40). Less than 5% had tried at least three types of triptans (Table 4).

**Table 4:** Distribution of the type of triptans experienced participants had tried.

Triptan type	Sumatriptan	Rizatriptan	Eletriptan	Frovatriptan	Naratriptan	Zolmitriptan	n
**Tried one triptan**							
	Yes						184
		Yes					31
			Yes				15
**Tried two triptans**							
	Yes	Yes					40
	Yes		Yes				10
	Yes					Yes	< 5
		Yes	Yes				< 5
	Yes				Yes		< 5
	Yes			Yes			< 5
**Tried three or more triptans**							
	Yes	Yes	Yes				9
	Yes	Yes	Yes			Yes	< 5

List of available triptans that triptan experienced participants had tried. No participants had tried Almotriptan. According to the study protocol, results of number of participants between n = 0–4 must be indicated as n < 5.

Seventy-four percent (N = 220) reported their level of function was improved to half of normal or more, and 82% (N = 243) reported a pain reduction of ≤ 50%, two hours post intake (Table 2). The pain reduction was significantly larger among triptan experienced participants who used triptans for their attacks than among triptan naïve participants who used common analgesic for their attacks, P < 1e−5 (Chi-sq), this difference was not significantly reflected by the level of function (P > 0.05). Stratified on pain reduction, only 16% (9/56) of the participants who had 50% or less pain reduction by triptan, had tried another type of triptan.

### Discontinuation of treatment with triptans

Of the 299 triptan experienced participants, 115 had discontinued their treatment with triptans at the time of interview. Reasons for discontinuation are listed in Table 5, and the main reasons were remission of migraine (43%) and insufficient effect (20%). Eleven percent (N = 13) discontinued treatment due to adverse events such as tiredness, dizziness, and heart palpitation. Eleven percent (N = 12) did not believe that their migraine attacks were severe or painful enough to take triptans. Anecdotally, participants also reported that it would be a hazzle to go to their GP to receive a prescription for triptans, since their pain was tolerable, and, thus, preferred to rest instead. This was not quantified. Of the participants who had discontinued their treatment with triptans, 65% were still using common analgesics.

**Table 5:** Discontinuation of use of triptans among triptan experienced participants.

Reported reasons	N (%)
Remission of migraine	50 (43)
Marginal or no effect of triptans	23 (20)
Adverse events	13 (11)
Believed attacks were too mild	12 (11)
Preferred other medication	9 (8)
Aversion towards medicine in general	< 5 (< 5)

Reasons for discontinuation of use of triptans.

N total = 115 of whom there were missing information on reason for triptan discontinuation on N = 6. According to the study protocol, results of number of participants between n = 0–4 must be indicated as n < 5.

## Discussion

We explored the use of triptans in a Danish population-based cohort. The main result was that there is a low adherence to the therapeutic guideline. Only 12% reported that they had been informed by their general practitioner (GP) to try the same triptan three times, before concluding that the triptan was ineffective. Furthermore, only 20% had been informed to try three different types of triptans, if the first or second triptan did not work. The majority of participants who had never tried a triptan (83%) had insufficient or no effect of common analgesics. Unfortunately, only 23% had been informed about triptans by their GP.

### Low adherence to the guideline of acute migraine treatment

Since 1994, a national guideline on the treatment of migraine, targeting GPs among others, has been regularly updated by the Danish Headache Society^2^. The guideline states that common analgesics sometimes combined with antiemetics are the first line of treatment. The second line of treatment consists of triptans. Treatment should be initiated as quickly as possible, and before concluding that the treatment is ineffective, the patient should try three different triptans, each during three different attacks^2^. Our results indicated, that triptan naïve participants with migraine had an unmet treatment need, since only 17% reported a significant effect of common analgesics on their migraine attacks.

Although not directly comparable, the reported effect of triptans was almost five times higher than the effect of common analgesics (17% vs 82%). Regarding level of function, this trend was marginally but not significantly different between the triptan naïve and triptan experienced participants (82% vs 74%). Thus, the low use of triptans was not compatible with the effect of common analgesics regarding the reduction of pain, but it was compatible regarding the improvement of level of function.

The end goal of acute migraine treatment is maximal pain reduction, preferably pain freedom, as quickly as possible and full function. According to the guideline, since only 17% of the participants reported sufficient pain relief by common analgesics, all triptan naïve participants should have been offered triptan treatment. However, only 23% had received information about triptans by their GP.

Likewise, triptan experienced participants were not adequately informed about triptans. Only 12% were instructed by their GP to try each triptan at least three times before changing treatment. Only 20% were informed that they should try two additional types of triptans, if the first two were ineffective.

It can be useful for individuals with migraine recurrence (i.e. relief of migraine following intake of acute migraine treatment with subsequent return of migraine within 24 h) to switch to another type of triptan for future attacks. Eletriptan and Frovatriptan appear to have relatively low recurrence rates^11,12^. The second dose of Sumatriptan is well tolerated and has been highly effective in treating migraine recurrence^13,14^. In the present study, three out of four participants were correctly instructed by their GP to take an additional dose of triptan, if their migraine recurred within 24 h.

Despite unmet treatment needs, less than 5% of the participants with migraine who reported low or no effect of triptans, had tried three or more triptans as recommended by the guideline. This was not because of economic reasons, since four of the available triptans are inexpensive due to generic substitution and public subsidize in Denmark (Sumatriptan: 0,5 $, Rizatriptan 1.4 $, Eletriptan 2.25 $, Zolmitriptan 2.7$ as of May 2021 according to publicly listed prices per dose (https://pro.medicin.dk). Furthermore, experiencing adverse events of triptans was not a major reason for discontinuation, since only 11% discounted triptans because of adverse events. This emphasizes that information about the safety and tolerability of triptans is important for patients, to ensure best possible adherence.

There may be many reasons why triptans underperform in clinical practice. Here, we report that one reason may be that the information and administration by GPs is suboptimal. Successful prophylactic treatment is no explanation, since use of prophylactic migraine treatment is low^15^. We would expect that the treatment in Denmark of migraine patients is optimal, since Denmark has free health care and the medicine is largely free. Thus, courses for GPs should focus more on the acute treatment of migraine.

### Low adherence of patients

Proper implementation of acute migraine treatment has the potential to improve patient outcomes^16^.

It has been reported that the most effective triptan is subcutaneous Sumatriptan with 80% response and 77% pain freedom after 2 h^17^. Subcutaneous Naratriptan has a similar or better response (88% pain freedom after two hours) but is not available today^18^. Even though subcutaneous administration is the most effective, 73% prefer tablets as first-route of administration^19^. Only 30% of the participants in our study discontinued triptans because of low efficacy or adverse events. This further indicates that triptans are well tolerated and effective, but paradoxically, only a minority of the general Danish migraine population repurchase a triptan^1^. It has been reported that only 29% of migraine patients experience pain freedom two hours after oral Sumatriptan^18,20^, also only 29% of migraine patients are very satisfied with their acute migraine treatment^19^. This may play a role in the low adherence to the guideline. Thus, the insufficient information by the GPs only explains part of the low adherence to triptans, observed in this and other studies^3^. Although not quantifiable, there was a general fear of triptans among the participants. The general belief was that triptans were stronger or more dangerous drugs compared with common analgesics. Most of the participants used common analgesics for their migraine, despite common analgesics having at least as many adverse events as triptans. Thus, it would be interesting to study triptan use in countries, e.g. in Sweden, where triptans are bought over the counter in contrast to Denmark, where prescription is necessary.

Even if patients only have few migraine attacks per year, the severity of the attacks causes high disability^21^. Migraine impairs or abolishes working capacity as well as social activity. And, fear of an attack highly impacts willingness to participate in social events^21^. Thus, even patients with few attacks should be effectively treated every time. Patients should be aware of the safety of triptans, hopefully leading to better adherence. The treatment can be optimized by patients who know the guideline. Even taking all the above into consideration, the present data suggest that it has been an overestimation to regard triptans as the ultimate solution to the acute treatment of migraine.

### Proposal for improvement

The results of this study indicate a need for more focus on education of the GPs regarding antimigraine treatment. Thus, courses given by headache specialists could ensure an increased knowledge of the treatment guideline. The courses would preferably be held on a regular basis to ensure that the newest guideline is followed.

Education and campaigns targeting the public about migraine and antimigraine treatment through a national public health program is suggested. It is important that the Danish government has funded a Knowledge Center on Headache Disorders at the Danish Headache Center. Its task is to spread knowledge of headache disorders among physicians and patients (https://videnscenterforhovedpine.dk/). The National Knowledge Center furthermore cooperates with patient associations for headache disorders in Denmark. This is important since patient associations help to inform migraine patients and their relatives about the disease.

A possibility would be to give easy access to three different types of triptan. Thus, a triptan package of 3 × 3 different triptans could be produced. The GP could easily inform the patient to take each triptan three times, and then make a choice for the future. Another possibility would be to allow triptan use over the counter. That would remove the erroneous belief that triptans are very strong and risky.

### Strengths and limitations

Our study is based on retrospective data which constitutes a risk of recall bias. A strength of the study was that participants were recruited from Danish Migraine Population Cohort (DaMP), which consists of participants with a validated migraine diagnosis (97% positive predictive value)^8^ and a known triptan status. DaMP is part of the Danish Blood Donor Study (DBDS) and largely representative of the Danish migraine population. Participants were easily reached through an electronic mailing system (e-Boks). The blood donors are generally dutiful people, thus, a high rate of them were willing to answer all questions as correctly as possible.

A limitation was that the random selection was done on in the DBDS. Hereby, the study does not show how many individuals with migraine in the general population are triptan naïve. This was, however, not within scope of the study. Furthermore, there are fewer participants with severe comorbidities in DBDS. We included 50 participants who had discontinued their triptan treatment because their migraine had ended. We note that their experience may not be as representative as the rest of the participants. We did not have information about medication overuse, since the risk of an overestimation of medication overuse among blood donors is very small, given the automated quarantine period if participants use analgesics of any form.

## Conclusion

In Denmark there is an insufficient adherence to the therapeutic guideline for acute migraine treatment. Only 23% of migraineurs were informed about the possibility of triptan treatment in the primary sector, when consulting their physician. Despite unmet treatment needs, less than 5% had tried three different triptans as recommended by the guideline. We suggest better education of the general practitioners to increase the standard of care. A public health program should educate migraine patients about their disease and its treatment. A package of 3 × 3 different types of triptans should be produced and prescribed to migraine patients. Finally, triptans should probably be delivered over the counter.

## Material and methods

### The Danish migraine population cohort

The Danish Migraine Population Cohort (DaMP) is a subgroup of the Danish Blood Donor Study (DBDS). DBDS is a large multi-center public-health study with an ongoing prospective cohort. Participants have been recruited from 2010 to 2020 from all regions of Denmark. Non-lactating and non-pregnant repeat blood donors between 18–67 years are invited to participate in the DBDS, and only 5% of the invited donors decline the invitation to participate^22^. N = 62,672 DBDS participants have previously answered a diagnostic headache questionnaire and were included in DaMP. The migraine diagnosis had a positive predictive value of 97%^8^. DaMP has been described in detail elsewhere and is representable of the general Danish migraine population in regards to migraine prevalence, triptan use, age, and gender-ratio, but with fewer chronic migraine cases and in general fewer comorbidities, such as uncontrolled hypertension^8^. Blood donors enter a quarantine period if they use analgesics of any form, thus the risk of an overestimation of medication overuse among blood donors is very small.

### Participants

We randomly recruited 400 participants with migraine from DaMP within two categories: Participants who had never tried a triptan (N = 101), i.e. triptan naïve, and participants who had tried and/or were currently using triptans (N = 299), i.e. triptan experienced. Participants within each category were contacted by telephone and interviewed by a specially trained senior medical student [AO] using a purpose-built semi-structured interview. The interview assessed adherence to or non-use of triptans and consisted of stratified questions for participants who were triptan naïve and participants who were triptan experienced. Triptan naïve participants were asked to further specify why they had never tried triptans, what information they received from their general practitioner (GP) about migraine and triptans, and if they were using other antimigraine treatment (here specified as paracetamol, ibuprofen, aspirin/caffeine combination and aspirin/codeine combination), prophylactic treatment, vitamins, alternative treatments such as acupuncture, osteopathic or chiropractic treatments. Triptan experienced participants were asked which triptans they had tried, how many times they had tried or purchased triptans, if they were current triptan users (N = 184), and if not, why they no longer used triptans (N = 115), details about information received by their GP regarding their migraine diagnosis and treatment guidance, the effect of triptans and the use and effect of other treatment as specified above. Flow chart of inclusion of study participants is presented in Fig. 1.

### Statistical analyses

Descriptive statistical analyses were used to study the association with triptan use and the assessed factors. We described the categorical variables as number of participants (n) and frequency (%). According to the DBDS protocol, results of number of participants between n = 0–4 must be indicated as n < 5. Statistical analyses were performed using statistical software R version 3.05 and R Studio version 1.2.1335.

### Ethics approval and consent to participate

Written informed consent was obtained from all participants. The study protocol was approved by the Danish Ethical Standards Committee (1-10-72-95-13, SJ-740, 1-90-09-88 and 1-70-04-07) and the Danish Data Protection Agency (P-2019-99) and conducted in accordance with the latest version of the Declaration of Helsinki.

## Supplementary Information

Supplementary Information.

## Data Availability

Data are available from the corresponding author upon reasonable request and requires both a material transfer agreement and memory of understanding in order to obtain ethical and data protection agency approval.
