# Supplementary material for: Low adherence to the guideline for the acute treatment of migraine
Source: Sci Rep. 2022 May 19;12:8487. doi: 10.1038/s41598-022-12545-2 (PMC9120453; doi:10.1038/s41598-022-12545-2)
Supplement: Supplementary file 1 — Supplementary Information. [file 41598_2022_12545_MOESM1_ESM.pdf]

Supplementary material

Supplementary Table 1. Other medication tried by triptan naïve migraine participants				
**Paracetamol**	**Ibuprofen**	**Aspirin/Codeine**	**Aspirin/Caffeine**	**n**
Yes	Yes	Yes	Yes	5
Yes	Yes	Yes	No	5
Yes	Yes	No	Yes	10
Yes	Yes	No	No	27
Yes	No	No	No	21
Yes	No	No	Yes	<5
No	Yes	No	No	<5
No	Yes	No	No	<3
No	No	Yes	Yes	<5
No	No	Yes	No	<5
No	No	No	Yes	<5
No	No	No	No	10

Supplementary Table 1: Other medication tried by triptan naïve participants. According to the study protocol, results of number of participants between n=0-4 must be indicated as n<5.
